# Community attitudes and gendered influences on decision making around contraceptive implant use in rural Papua New Guinea

**DOI:** 10.1186/s12978-020-00985-w

**Published:** 2020-09-05

**Authors:** Sarika Gupta, Sarah Bernays, Kirsten Isla Black, Philippa Ramsay, John Bolnga, Angela Kelly-Hanku

**Affiliations:** 1 Discipline of Obstetrics, Gynaecology and Neonatology, Central Clinical School, Faculty of Medicine and Health, The University of Sydney, Sydney, New South Wales 2006 Australia; 2 Department of Global Health and Development, London School of Hygiene and Tropical Medicine, Bloomsbury, London, UK; 3 Department of Women’s Health, Neonatology and Pediatrics, Royal Prince Alfred Hospital, Missenden Road, Camperdown, New South Wales 2050 Australia; 4 Department of Obstetrics and Gynaecology, Modilon General Hospital, Modilon Road, Madang, Madang Province Papua New Guinea; 5 Sexual and Reproductive Health, Papua New Guinea Institute of Medical Research, Goroka, Papua New Guinea; 6 Kirby Institute for Infection and Immunity in Society, University of New South Wales, Sydney, New South Wales 2052 Australia

**Keywords:** Long-acting-reversible-contraception, Contraceptive implant, Decision-making, Gender, Inequality, Rural Papua-new-Guinea

## Abstract

**Background:**

Despite targeted interventions to improve contraceptive implant acceptability and uptake in rural Papua New Guinea (PNG), ongoing use of this method remains limited. Previous literature has suggested community attitudes and intrinsic factors within the decision-making process may be negatively impacting on implant uptake, however these elements have not previously been studied in detail in this context. We set out to explore community attitudes towards the contraceptive implant and the pathways to decision making around implant use in a rural community on Karkar Island, PNG.

**Methods:**

We conducted 10 focus-group (FGD) and 23 in-depth interviews (IDI) using semi-structured topic guides. Key sampling characteristics included age, exposure or non-exposure to implants, marital status, education and willingness to participate in discussion. Four FGDs were held with women, four with men and two with mixed gender. IDIs were carried out with five women (current implant users, former implant users, implant never users), five men, five religious leaders (Catholic and non-Catholic), four village leaders and four health workers. Two in-depth interviews (four participants) were analysed as dyads and the remaining participant responses were analysed individually.

**Results:**

Men were supportive of their wives using family planning but there was a community-wide lack of familiarity about the contraceptive implant which influenced its low uptake. Men perceived family planning to be ‘women’s business’ but remained strongly influential in the decision making processes around method use. Young men were more receptive to biomedical information than older men and had a greater tendency towards wanting to use implants. Older men preferred to be guided by prominent community members for decisions concerning implants whilst young men were more likely to engage with health services directly.

**Conclusions:**

In communities where a couple’s decision to use the contraceptive implant is strongly coloured by gendered roles and social perceptions, having a detailed understanding of the relational dynamics affecting the decision-making unit is useful in targeting future healthcare interventions. Engaging groups who are reluctant to connect with health information, as well as those who are most influential in the decision making process, will have the greatest impact on increasing implant acceptability and uptake.

## Plain English summary

Contraceptive implants are a reliable and reversible form of birth control which can effectively reduce the number of women who die from complications associated with childbirth. Papua New Guinea is an island nation in the Pacific region in which high numbers of women continue to die in childbirth but where very few women are using implants. In these regions, complex factors influence a woman’s decision to use birth control, as well as the type of method she chooses to use. We used an interview based design to study in detail the decision-making processes surrounding the use of the contraceptive implants and focused on how men and women interact with one another and their communities within this process. We centred our study in one rural community on Karkar Island, Papua New Guinea and discovered that, concerning the use of implants, men were more influential in the decision making process than women. All members of the community reported the need to feel as though they could ‘trust’ a method before they used it and most persons did not know enough about the implant. Older men were reluctant to learn about the implants from health workers, preferring to liaise with their village leader or religious leader. In contrast, younger men were more open and pro-active to learn about implants by approaching health services directly. These findings may help to guide health planners who are hoping to expand access to contraceptive implants in rural communities throughout Papua New Guinea.

## Introduction

The acceptability and uptake of long acting reversible contraception in many low and middle-income countries (LMIC) is shaped by a complex interplay of factors including organizational logistics, challenging geography, sparse human service resourcing issues and a lack of integration and understanding of the diversity of traditional knowledge and practices around reproductive health [1–4]. Qualitative research from these diverse settings also highlight the broader gendered issues which underpin key differences in men and women’s attitudes towards reproductive health and impact upon uptake of the various contraceptive methods available [4–9]. While research around the use of modern contraception in LMICs is expanding [1–3], the existing literature on how gender, as a relational issue, impacts upon the decision-making process to accept long acting reversible contraception is in its infancy. This is especially the case in the highly gendered setting of Papua New Guinea (PNG), the focus of this paper.

PNG is a culturally diverse archipelago nation in the Pacific and has one of the highest fertility rates and one of the lowest modern contraceptive prevalence rates globally [2, 3]. A predominately rural population (> 85%), the actual fertility rate in rural settings has been consistently higher than the reported desired fertility rate for the past decade [10]. The unmet need for contraception in these areas persists at 34.2% with long acting reversible contraception only making up 2% of in-use methods [10–13]. There is a growing body of evidence in support of long acting reversible contraception, and in particular the contraceptive implant, becoming the preferred method of contraception in LMICs given their high efficacy rates, cost effectiveness, mild side effect profile, low complication rate, non-user-dependent administration, minimally invasive design and technical simplicity for insertion and removal [2, 14, 15]. Since 2013 there have been concerted efforts to improve access of contraceptive implants across PNG via outreach programs led by local health authorities and non-government organisations [2, 16].

In one rural community on Karkar Island outreach programs were expansively conducted between 2013 and 2014 [16]. These programs successfully increased implant uptake on the island and boosted contraceptive use by 12% [11, 16]. However, despite 12 month follow up data indicating high satisfaction rates amongst women who were using the implants, and 90% of women reporting an intention to continue using such a method in the future, implant use on Karkar Island has fallen by 30% since 2016 [11–13, 16]. Interestingly, modern contraceptive prevalence rates on the island did not fall by the same degree, suggesting women were using other modern methods instead of the implant [11–13]. Service constraints and resource limitations may account for part of the observed reduction in implant use, but given the complex socio-cultural setting of PNG, there is a need to explore the ways in which the uptake of implants is influenced by these factors, including gender.

The societal structure of most communities in PNG is innately gendered [17]. Men play a key role in family planning decision-making which stems from long-standing beliefs around the societal roles of men, women and children [17]. Early data describes the nuclear family as the basic unit of society which is typically organised around a ‘big man’ [17, 18]. A ‘big man’ is prestigious within his community; he is able to gather supporters around him, he functions as the head of the family and he is the final arbiter of decisions [17, 18]. Historically, and still in many parts of the country today, the societal value of women lies in their capacity to produce and raise children for their husband’s clan and therefore the wealth they will attract for their paternal families in pigs and garden food that would determine their ‘bride price’ at the time of marriage [17–19]. Marriage is therefore an important pathway for men to gain prestige within their communities. Without marriage and reproduction, for which they are dependent on women, their ambitions to become ‘big men’ is severely curtailed [17–19].

Introduction of modern contraception into PNG in the 1980s to limit family size and delay childbearing for other pursuits such as education, challenged these traditional paradigms and dissociated both men and women from their cultural roots and heritage [17–20]. Men in particular reported feeling incompetent because service providers failed to consult and involve them in the development of reproductive health campaigns [20, 21]. Combined with traditional perceptions that menstrual blood, particularly after child-bearing, is considered ‘dirty’ and ‘dangerous’ with the potential to cause ‘weakness’ in men, this resulted in an alternative social consensus emerging in which exposure to and awareness of reproductive health became recognised as the preserve and responsibility of women whilst men were better posited to attend to economic and financial matters [17, 20, 21]. These social attitudes were found to persist in more recent studies from the highland provinces of PNG in which men acknowledged the value of family planning, but they continued to perceive it as ‘women’s business’ [22]. Importantly though, their perception did not disqualify these men from being the final decision makers around the method of contraception used by their wives [22].

In order to explore the issues of gendered relationships, community perceptions of family planning and pathways to decision making around method use we undertook a qualitative study to understand these issues in one rural setting in PNG: Karkar Island. So as to inform future program planning we specifically investigate how community attitudes towards the contraceptive implant reconcile with gendered relations within the decision-making process and how these may be impacting on women and men’s decisions around implant use.

## Methods

### Research setting

Karkar Island is a rural community off the coast of Madang town, Madang Province on the north coast of PNG, and is a two hour boat ride from the mainland. In 2016 the population was estimated at 60,000 with 31,200 females (52%), of whom 51% were in the reproductive age range of 15—49 years [11, 23]. Less than one in three (29%) of women of reproductive aged were using modern contraceptives in 2016 [11, 12]. Amongst those using a modern method, 40% were using implants, 31% were using injectables, 31% were using the oral contraceptive pill and the remainder were using condoms, had had a tubal ligation or their husband had had a vasectomy [11, 12].. Implant uptake amongst women on the island since 2016 has been minimal [23].

There are 52 villages on the island of which 41 (79%) are along the island’s 84 km coastline [11, 23]. Villages are connected by a continuous road which is subject to flooding. The island is serviced by one district hospital, two major health centres and 23 peripheral aid posts which are all accessible by road. The hospital and health centres are continuously staffed by nurses, midwives, doctors and community health workers whilst staffing at aid posts is inconstant. Family planning counselling and services should theoretically be available from all sites [23].

The socio-demographic make-up of Karkar Island is similar to other rural communities throughout Papua New Guinea because there is a high proportion of reproductive aged women, an increasing number of adolescents, a strong religious presence in the community, the majority of families rely on subsistence income and population literacy rates are low [11, 16]. However the unique geography of the island means that women have greater access by road to the major health facilities; because of this engagement with antenatal services and the number of supervised birth rates on Karkar is between 15—30% higher than the rural national average [11]. The population of Karkar Island is also relatively isolated from the mainland which minimizes the effect of shifting populations on the location’s health profile.

Each village on Karkar Island is headed by one or two leaders, a church representative and four to five family elders, all of who are typically men [11, 23]. Social ranking is determined by age, gender and land asset with village heads responsible for maintaining order within and between villages, including resolving family and marital disputes where necessary [11, 12]. There is little in the way of formal employment on Karkar Island with almost all men and women reliant on subsistence agriculture or informal markets for their livelihoods [11, 12].

### Study design, participants and recruitment

The data used in this paper was drawn from a sub-set of a larger mixed methods study on the impacts of contraceptive implants on maternal and neonatal health [16, 23]. As part of the qualitative study, focus group discussions (FGD) and in-depth interviews (IDI) with community members and healthcare workers were used. The purpose of the qualitative sub-study was to explore community attitudes towards the contraceptive implant and the pathways to decision making around the use of the implant.

Twelve coastal and four inland villages were randomly selected for sampling using a computer-generated ballot. The research team liaised with the leaders in each village and together they invited participants to partake in FGDs. Snowball sampling was then used to invite men and women to partake in IDIs (Table 1). Key sampling characteristics for participants included age, exposure or non-exposure to implants, marital status, education and willingness to participate in discussion. We classified young people as those persons under 25 years of age according to the World Health Organisation definition [24]. We used a dyadic approach with the two couples who agreed to be interviewed separately and were aware that their accounts would be analysed alongside their partner’s to directly compare the two perspectives within the same couple unit. Interviews for members of each couple unit were unable to be carried out simultaneously but were carried out successively without opportunity for them to convene and discuss with one another [25].

**Table 1:** Focus-group discussion and in-depth interview participant groupings

Interview Type and Participants	Characteristics	Number of Interviews
**Focus Group Discussions**		**10**
Men	Mixed age	1
	Age < 25	1
	Age > 25	2
Women	Mixed age	1
	Age < 25	1
	Age > 25	2
Men and Women	Mixed age	2
**In-Depth Interviews**		**23**
Men	Partner implant user	2
	Partner implant former- user	1
	Partner implant never-user	2
Women	Implant user	2
	Implant former-user	1
	Implant never user	2
Village Leader	Coastal village	3
	Inland village	1
Health Worker	Nurse	3
	Village health volunteer	1
Religious Leader	Catholic	3
	Non-Catholic	2

### Data collection

A semi-structured topic guide was used to guide the FGDs and explored the following areas: role of family; family planning knowledge; family planning perceptions; experience with the implant; decision making around implant use; and potential enablers and barriers towards implant use. Prior to conducting the FGDs the topic guide was informed by formative work with the research team to ensure its contextual suitability.

The development of the topic guides for IDIs was informed by iterative interim analysis of FGD data. IDIs explored the decision-making processes around implant use and non-use in detail including: why implants are used or not, who plays a role in the decision making process, who provides advice to women, who provides advice to men, what actual experience of implant use has been, why women stop or discontinue implant use and what personal and community attitudes are towards unintended and teenage pregnancy.

All FGDs and IDIs were audio-recorded with participant consent and later transcribed and translated from Tok Pisin to English by independent researchers. IDIs lasted an average of 55 min. Not conversant in Tok Pisin, the lead author recruited a Papua New Guinean researcher trained in qualitative research to conduct the IDIs and FGDs. The Papua New Guinean researcher was provided training by the lead author on the research tool and the aim of the study. The lead author met with all participants and thanked them for their involvement but was only present in the FGDs and only participated in and ad-hoc manner in IDIs as the lead interviewer shared information in English. A male health care worker known to the community supported the lead interviewer to ensure men were comfortable being interviewed by a Papua New Guinean woman.

### Data analysis

Written transcripts were analysed using thematic analysis following the models described by Neuman and Silverman [26, 27] whereby transcripts were read and re-read in a process of familiarisation. They were then open-coded using techniques outlined by Strauss and Corbin [28]. A coding framework was then developed and applied to the data. Analytical memos drawing on coded material supported the process of charting to cluster coded data into groups and categories to develop the main themes which described and characterised the primary findings from the transcripts. We used methods of triangulation to compare findings from within the same couple unit, across gender and age within the interview and FGD data and then cross-validated these findings with community leaders to enhance the richness of the data and to be able to account for variation in perspectives [29, 30].

## Results

### Study participants

The characteristics of the 89 study participants are outlined in Table 2. The age range was from 17—52 years with a mean of 31 years. Fifty-seven percent of respondents were women. Of the male respondents, 41% were young men (< 25 years). Almost two thirds of all respondents started primary school but three-quarters of this group did not complete Grade 7. Amongst young people, a larger proportion were educated to secondary level or higher (51%) than older people (18%).

**Table 2:** Background characteristics of study participants (*N* = 89)

Characteristic	Number	Percentage (%)
*Gender*		
Men	38	43
Women	51	57
*Age*		
< 25 years	32	36
> 25 years	57	64
*Education*		
None	24	27
Elementary and primary	53	60
Secondary or higher	12	13
*Literacy*		
No literacy	64	72
Literate	25	28
*Occupation*		
Subsistence farmer	54	61
Casual employee	8	9
Village leader	9	10
Church worker	14	16
Skilled worker	4	4
*Religion*		
Roman Catholic and Lutheran	45	51
Other	4	4
*Number of children*		
Less than 3	38	43
3 or more	51	57

### Community attitudes towards family planning and implant use

The majority of participants, including religious and village leaders, acknowledged and understood the need for family planning in order to limit family size as well as space a woman’s pregnancies. Land and housing shortages as well as financial issues on the island have very real consequences for couple’s decision making as well. Many couples interviewed spoke of only wanting up to three children.

*‘Three children is enough to help us with the land and look after us when we are old but it is not too much for my husband to pay for their schooling and for their food and clothes. I am also happy with three because I can love them equally.’* **Non-implant user, aged 26.**

There was also a recognition by both women and men, including religious and village leaders, for a woman to have adequate spacing between her pregnancies to ensure the health of the expectant mother and by investing in this, the overall health of the unborn/newborn baby. With a healthy mother and baby, the emotional and physical well-being of the whole family benefits.

‘*It is important for mothers to have time to recover their health after birthing otherwise their body becomes weak. If the mother is weak she cannot feed the child, she cannot look after things in the house and she cannot care for her husband properly. All of these things are important for the husband to be able to do his duties without worry.*’ **Village leader and husband of implant user, aged 32.**

*‘I had two pregnancies close together and with the last one we had twins. This was very difficult on my body. I had to send my eldest child to my sister’s village so she could care for him while I tried to look after my babies. Sometimes when the babies were sick my husband would have to stay home from work to help me so I could tend to the house; this made him angry but we had no choice. It was very hard for us.’* **Non-implant user, aged 24.**

There was an overriding preference amongst all respondents to use family planning methods which they felt comfortable with and ‘trusted’. Trust was critical to a couple’s understanding and acceptance of family planning methods and the contraceptive method chosen. Healthcare information was ‘trusted’ if it could be corroborated through discussion with village peers. Information about family planning, like other important health issues in Papua New Guinea, is assembled by different types of information from a variety of sources. For example, in addition to biomedical knowledge, other forms of knowledge are derived from cultural and religious domains. Biomedical and social knowledge and information becomes more valid if these messages are seen to be consistent. For women, healthcare information becomes socially validated through discussion with other women, sisters, aunts, mothers and infrequently with church leaders. Whilst for men, validity emerges through dialogues with friends, fellow workers, older family members, village elders and religious leaders.

*‘I talked to my wife about family planning and her preferences. She did not like the pills anymore and wanted to try the implant but she could not answer my questions about side effects so I spoke with my cousin whose wife was using Depo and she was happy without complaints. So that is why we went for the Depo. As the leader of the house I feel responsible to make the right decisions for my family. If I do not have enough information I do not feel like I can make a good decision so I have to find more information myself. I trust the information from my family and elders.’* **Husband of non-implant user, aged 27.**

Most respondents reported familiarity with and therefore ‘trust’ in the combined oral contraceptive pill, Depo Provera injections and condoms. Conversely, there was a strong undercurrent of fear and apprehension towards the implant, or ‘rubber’ as it was locally called. Little was known about the mechanism and side effects of the implant which ultimately lowered community confidence in the device and prompted women to use alternative known and trusted methods.

*‘I am scared to let my wife use the rubber in case it makes her sick and then she will not be able to do her duties in the house. Me and my family do not know enough about it. It is better not to take a chance with things we do not know about.’* **Husband of non-implant user, aged 30.**

Health-workers, particularly those with informal training, felt poorly positioned to alleviate community concerns and questions about the implant either because they lacked the depth of knowledge themselves, or if they do, they felt unable to compete with societal beliefs and concerns.

*‘Us health workers struggle to defend the implants at times. Men and women are scared to use it because they do not know much about it and they do not trust it. Even if we show them it is safe, they do not believe us because their friends and family convince them not to use it.’* **Female community health care worker, aged 29.**

### Socio-cultural factors impacting upon implant awareness, acceptability and uptake

#### Intrinsic factors influencing the decision-making process

**Fig. 1:**
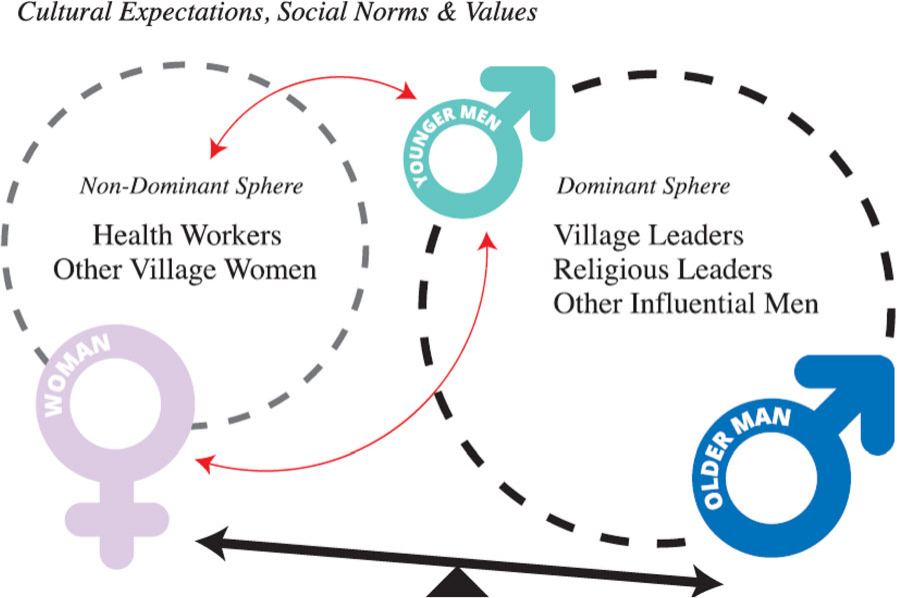
Spheres of influence affecting men and women in reproductive healthcare decision-making

Longstanding societal norms dictate that men have a stronger power of influence than women within household decision-making, including those concerning reproductive healthcare. The process of decision-making between man and woman in the couple unit is relational and is substantially influenced by distinct external spheres which are infused with gendered characteristics. One sphere contains village leaders, church leaders and other influential men: the direct audience for this sphere is the man and owing to their power of influence within the decision-making process, this goes on to represent the dominant sphere (see Fig. 1). The other sphere consists of health care workers and other village women: the direct audience for this sphere is the woman.

Women receive information about implants from health care workers during clinic visits and from other women during their day-to-day interactions. Women then recall this to their partners. In turn men reflect on the information remembered and shared by their wives before consulting other members within their direct sphere of influence and coming to a final decision on whether his wife should or should not have the implant. In this way women function as conduits of information but in no way do they expect to be the sole decision-maker about implant use.

*‘Us women are not allowed to make decisions that affect our body without consulting our husbands and getting their approval, especially not for new methods like the implant. It does not matter what the law says; this is our law and we abide to it because if we do not, there will be disputes’* **Implant user, aged 39.**

Importantly, the spheres were not observed by researchers to communicate with each other and the health care worker who is expected to be the most knowledgeable about the implant and represents the ‘source of biomedical information’ is not contained within the dominant sphere. This exclusion is more likely to have a negative impact on implant uptake because general community knowledge of the implant is low and health care worker knowledge becomes relatively uninfluential in dispelling mis-information about the device within the dominant sphere. An emerging subset interaction between young men and health care workers may balance transfer of biomedical information into the dominant sphere and this exchange will be explored in more detail later.

#### Male perceptions of reproductive health

There is a deeply engrained perception that family planning is ‘women’s business’ and it is perceived by men to be of less value than economic or financial concerns. Prioritisation of economic matters is driven by a state of poverty in which men feel they would compromise their family’s wellbeing if they were to redirect their attention towards reproductive healthcare matters. This explanatory framework, which posited that men prioritised economic concerns, was generally cited as the justification for their limited attendance at implant counselling sessions and their lack of motivation to directly liaise with health care workers to learn more about the method.

*‘Family planning is women’s business. They are the ones to learn about it from their mothers and sisters and the sisters in the clinic. My responsibility is to provide for my family by tending to the land. It would take me away from the crops for one whole day to go to the clinic and then wait for the health worker to discuss. We do not have the luxury of this in my family.’* **Husband of implant user, aged 28.**

*‘It is my priority to generate finances to feed and house my family. I do not have time to attend such things as women’s business and family planning. It is not my interest.’* **Husband of non-implant user, aged 30.**

More recently, some outreach campaigns have tried to include men in implant counselling [16, 23]. Incorporating men into the interaction between women and health-workers essentially draws them into an alternative sphere which they do not readily interact with (see Fig. 1) and for some participants this was described as uncomfortable and potentially emasculating. Consequently, there is often an active resistance for men to interact with health workers, particularly female ones, out of fear of social judgement.

*‘In my community it is considered weak if a man is involved in women’s business. I would lose the respect of the other men in my village and this would upset me.’* **Husband of non-implant user, aged 39.**

There has been limited attention paid to bringing the health care worker into men’s dominant sphere of influence which in turn limits the transfer of biomedical reproductive healthcare knowledge into this sphere. Information transfer is further restricted because most healthcare workers are women and as such they are unable to approach men directly to enter their dominant sphere of influence because it breaches the accepted gender norms of the community.

‘*As a female health worker I cannot approach a male directly without his wife. It is disrespectful; especially if I am wanting to talk about intimate topics like family planning.’* **Midwife, aged 30.**

Men though, especially those who lived in close proximity to the health centres, were more likely to be receptive towards older male health workers and would more readily invite them to liaise with social influencers including village and church leaders. The impact of this was diluted though by the majority of male health workers being informally trained which limited their capacity to counsel about implants.

*‘I am an older male and well respected by my community. I learned about family planning from a workshop run by the church and I was given permission to pass this knowledge onto the other members of my community. I often speak with our village leader about the importance of spacing pregnancy and he agrees with this. But I only know some things about pills and depo, not much about the implant, so I cannot answer all of their questions.’* **Male village health volunteer, aged 43.**

#### Women driven factors

Biomedical information concerning the safety and side effects of implants is readily imparted between healthcare workers and women because rapport and connection is enhanced by their shared gender. Women then disperse these health messages through the community via discussions with friends and family. Whilst this can be a rapid and effective pathway for information transfer, these pathways also give rise to second and third hand information which can result in rumours and mis-information about the implant; particularly amongst those women who have poor literacy or live remote from the major health centres where access to health information is greatest. Due to the intrinsic need for information to be corroborated with peers and family members, these rumours have the potential to challenge the credibility of biomedical messages and impact on the quality and accuracy of information which is eventually passed onto men. Amongst some women residing remotely from health centres, rumours concerning the implant centred on the debilitating side effects of the implants and the notion that implants have been introduced by foreigners to spy on the locals. In a community where trust is paramount to the acceptance of information, these rumours have been observed to fracture women’s own receptivity towards using the device, independent of their partner’s impressions.

*‘I trust what the health worker tells me about the implant because she is a good lady. But my sisters and mother have heard stories from other women they know who say the implant is not good and I cannot ignore their advice.’* **Implant non-user, aged 27.**

*‘My friend told me the implant has a camera inside which sends your information outside to Moresby and other countries. When I heard this I went and had it taken out’* **Implant ex-user, aged 34.**

*‘I have heard the implant makes your body weak. I know someone who used it and now she cannot move her arm even after it was taken out. I do not want to use such a thing in my body’* **Implant non-user, aged 29.**

### Changing of the baton from old to young

There were important differences in attitude and relational behaviour between young people and older participants. Younger men were considerably more open to discuss implant use with their partners and to engage with the health workers directly to seek out biomedical information (Fig. 1). This is illustrated in the differences identified between the two couples whose interviews were analysed using a dyadic approach. The woman from the older couple was comfortable to defer family planning decisions to her partner. In contrast, the younger woman engaged her husband in a discussion about the implant and invited him to liaise with a male health worker to gain more information. This pattern was reflected across the data suggesting a changing social attitude amongst younger participants.

*‘Us young men are the next generation of our country. We cannot be ignorant to what is happening around us like some of our fathers are. They are scared of knowledge but we are not. We crave it. I do not know very much about family planning because it is not taught to us in school but we can sometimes find information on the internet. If there is something we do not understand then we can talk to the health workers about it and they usually help us.*’ **Man, aged 19.**

Although there were some older married men who recognised the need for husbands to adopt a more pro-active approach to seek out information and move towards informed decision making around implant use, they were the exception. This tended to be driven by specific health and economic circumstances in their family.

*‘I was scared we would have another child close together and we would not be able to care for them. This was my motivation to learn about family planning and the implant because I had heard rumours it was very effective but did not know enough about it. Some of my friends laughed at me but it was OK because I knew I could not afford more children so I did not care for their views.’* **Husband of implant user, aged 36.**

*‘Sometimes I wish I had more courage to defend my wife’s wishes. Her body was affected by TB but in our culture it is very important to have a male child to inherit the land and the title. We have three daughters so we need to keep trying for a male. This is why I am not allowing her to use family planning.’* **Husband of non-implant user, aged 29.**

Overall, while this changing pattern was acknowledged, many older men thought that it was a shift that threatened the dominant order and ought to be resisted.

*‘The young men of today are too confident. They do not care for our values and traditions. Our elders have been guiding our families for generations. It is foolish to dismiss their advice for the sake of something they read on the internet. There is a place for education and information but not at the expense of our elder’s knowledge.’* **Man, aged 28.**

What emerged from this data was a ‘transitional’ model in which a new sphere of influence is developing between young men and women that is more inclusive of health care worker knowledge. This emerging relationship establishes a conduit through which biomedical information concerning the implant can enter the dominant sphere of influence which then has the potential to lead to more informed decision-making around implant use because young men are able to take first-hand information from health workers into their discussions with community influencers.

## Discussion

Our data illuminates a number of important themes and sub-themes which are intrinsically linked. First, we identified that men on Karkar Island are keen to support their wives to use family planning but there is a lack of community understanding into the contraceptive implant in particular which drives men and women to opt against using the method. Second, though men perceive family planning to be ‘women’s business’, they remain strongly influential in the decision making processes around which methods of family planning their wives eventually choose to use, and more importantly, their investment in remaining influential in this process stems from long-standing cultural expectations of men and women’s societal roles. Third, there was a multifactorial dis-interest among older men to increase their knowledge and awareness around contraceptive implants but we discovered this attitude to be shifting amongst young men into one that was more inclusive of and receptive to reproductive health information.

### Community awareness and support for using contraception

Support for family planning interventions on Karkar Island is dichotomous in its ability to space pregnancies and limit family size. Preserving women’s health by enabling them to space their pregnancies is viewed as an investment in the broader health and wellbeing of the family unit and ultimately the community [17, 18]. If a woman is healthy, she is better able to rear her children and support her husband to become a ‘big man’ which eventually enhances the broader social hierarchy [17–20]. Limiting family size to have three children means that couples can meet the socio-cultural obligations for reproduction without putting unreasonable economic pressure on the family unit [20]. Our data confirms that both women and men on Karkar Island clearly recognise the value of modern contraception in achieving adequate pregnancy spacing and limiting family size but there was a lack of acknowledgment for contraceptive implants to be the most preferred or effective method.

Prior to the implant program Depoprovera (‘Depo’) was the most commonly used method of contraception on the island [11]. While Depo has the advantage of less irregular bleeding, our research shows that women were accepting of the irregular bleeding with implants and while 25% had this side effect, only 2% of these women discontinued use for bothersome bleeding at 12 months [16]. This may be because the personal and social consequences of having an unintended pregnancy are becoming more significant for women in PNG and despite cultural perceptions that menstrual blood is ‘dirty and dangerous’, individuals and communities are beginning to re-shape their perceptions around irregular bleeding in preference for effective and reliable contraception [31, 32]. The key advantage of the implants over Depo is that they are long lasting (reducing clinic visits and supply chain issues) but quickly reversible [2].

### Decision-making around contraception

According to the traditional social architecture of PNG and most other Melanesian societies, men and women function as dividuals rather than individuals [33]. Dividuals are sociocentric as opposed to individuals who are egocentric [33]. Decisions and actions of men and women within dividual settings are therefore not autonomous but heteronomous as determined by their relationships with each other in the couple unit and with their families and wider community [33]. Furthermore there is a long standing and rigid role dichotomy between men and women in PNG which results in men having a stronger seat of influence within the decision making process than women [17–20, 33, 34]. Christian missionisation throughout PNG brought new ideas concerning gender by focusing on the nuclear family as the basic unit of society rather than the traditional extended family posited around a ‘big man’, but in itself this new value system has not overridden traditional approaches to decision making around family planning [17, 20, 33, 34]. Findings from our data confirm that decision making around family planning methods on Karkar Island remains heavily coloured by the various social spheres of influence (Fig. 1) and that deeply entrenched gendered perceptions allow men’s direct sphere of influence to be more powerful than women’s within this process. Improving implant uptake is therefore dependent on enhancing the receptiveness of the men’s direct sphere of influence to accept information about the device [20, 34].

The rigid role dichotomy between women and men contextualises why family planning awareness and knowledge is perceived as ‘women’s business’ but does not entirely account for why community awareness and acceptance of implants remains low. Biomedical information only represents part of the source of information [34]. There is substantial scholarly work from PNG which details the critical importance of religion in making sense of disease and treatment in culturally relevant ways and the impact this has on community acceptance of health care interventions [35–37]. Christianity is the dominant religion in PNG and churches are major providers of health and education services but these organisations are not actively involved in expanding implant awareness on Karkar Island at present; it remains the preserve of health centres and clinics [16, 23, 35].

Moreover it is likely that religion influences the work and practices of most healthcare workers, not just those employed by faith-based organisations [35]. The church’s inactivity in promoting implant use on the island may be colouring the information being delivered about the implants by some health care workers and contributing to the ongoing circulation of rumours. As suggested by studies from other rural provinces in PNG, it could be more effective to expand community awareness and acceptability of implants by engaging religious leaders as advocates for implant use [20–22, 34]. This would directly engage the dominant sphere of influence whilst enabling healthcare messages about the implant to be delivered via a conduit that the community recognise, respect and culturally accept [20, 34].

Men’s general disengagement with biomedical health information can be explained by their preference to value economically incentivised messages over health messages alone [20, 22, 34]. Men in our focus groups stated economic disruption as the main reason for not engaging with health services. Re-framing the benefits of limiting family size and spacing pregnancy in remunerative rather than gendered terms may help to shift the discourse of men to engage with health care messages [22]. Engaging village leaders, patriarchs and other male influencers to incentivise the pecuniary value of contraception has shown promise in increasing method uptake amongst married and unmarried couples in other low and middle income countries, most notably in rural India and Nepal [6, 38, 39] and may be another strategy for engaging the dominant sphere of influence on Karkar Island. Delivering biomedical health information via trained male health workers who can liaise directly with men in their homes would also allow men to receive information without distracting from their economic responsibilities [40, 41]. Such schemes have improved spousal communication and ultimately led to increased contraception uptake in sub-Saharan communities [40–42].

The emerging attitude shift in the way young men and women engage with health information in our study, which ultimately filters through to their decision making choices around implant use, is inspired by a recent, global and rapid change in young person’s access to diverse external information via the internet and social media [43, 44]. Such altered exposure provoked the younger men in our study to proactively seek out biomedical information from health workers to inform their decisions and in doing so by-passed the more inflexible pathways that determine information access and acceptability for their elders. By contrast, older men and women in the community remain relatively isolated from modern sources of information because they reported a fear of criticism from their peers if they were to access sexual and reproductive health resources via the internet or mass media. This mirrors findings from other similarly conservative Polynesian and South Asian communities where there is a tendency among older persons to remain sceptical of information from external sources until it were accepted and trusted by community influencers [45].

International data confirms there is an evolving preference amongst young persons for implant use due to their long action and user independent profiles [46, 47] though these findings are yet to be reported amongst young persons in PNG. Bell et al. (2018) identified a lack of specific reproductive health services for young persons in PNG as an important barrier in encouraging them to engage with family planning on an ongoing basis. Youth specific services would be particularly beneficial to protect young people from the aforementioned preconceptions of their adult peers and if included in future programs, these services may help to enhance implant uptake among young people [48].

In isolation these interventions are unlikely to achieve significant shifts in the intractable gendered dialogues that influence decision making around family planning on Karkar Island and similar communities throughout PNG. Instead, enhancing education for young people is a broad and powerful strategy for improving community health because it helps to foster positive health seeking behaviours from a young age which then become trans-generational [49]. The Global Strategy for Women’s, Children’s and Adolescents’ Health (2016—2030) in line with the Sustainable Development Goals 2030 Agenda are focusing on retaining adolescents in school and relying on the associated improvements in literacy to enhance their understanding of contraceptive benefits [49, 50].

#### Limitations

This study analyses decision making dynamics amongst a single community in response to a retrospectively introduced intervention and our observations may not be transferrable to other populations without further research. A major pre-requisite for men and women to develop greater interest in biomedical information about implants rests on the assumption that current perspectives amongst young people remain unchanged and do not become indoctrinated by changing social roles over time. Using dyad interview techniques provided richness to our analysis around shared decision making but our ability to represent the data using direct quotes was challenged by the well described ethical constraints of exposing participant identity [51]. Importantly our study findings are based on participant recounts rather than observed behaviours and we should be circumspect in interpreting these findings and extrapolating their longitudinal impact.

## Conclusions

In communities, such as on Karkar island, where a couple’s decision to use the contraceptive implant is strongly coloured by gendered roles and social perceptions, having a detailed understanding of the relational dynamics affecting the decision-making unit is useful in targeting future healthcare interventions. In the short to mid-term recruiting respected community members such as religious and village leaders to be health messengers, and reframing the benefits of implants in economic terms will likely have a catalytic effect on engaging older men with reproductive health services, which may ultimately encourage method uptake. Simultaneously, if health strategists cultivate the pro-active information seeking attitudes evolving amongst young persons by developing specific reproductive health services for them, this will pave the way for the new ethos of shared and informed decision making to emerge as a longer term solution to increasing implant uptake.

## Data Availability

Data sharing is not applicable to this article as no datasets were generated or analysed during the current study. All interview and focus group discussion manuscripts are available from the corresponding author in raw and coded form on reasonable request.

## Abbreviations

PNG: Papua New Guinea
LMIC: Low and middle income countries
FGD: Focus group discussions
IDI: In depth interviews
